# Economics and industry do not mean ethical conduct in clinical trials

**DOI:** 10.1186/2052-3211-6-11

**Published:** 2013-12-02

**Authors:** Joel Lexchin

**Affiliations:** 1 School of Health Policy and Management, York University, 4700 Keele St., Toronto, Ontario M3J 1P3, Canada

**Keywords:** Clinical trials, Developing countries, Ethics, Ghost writing, Pharmaceutical industry, Seeding trials, Withholding data

## Abstract

Clinical trials present an ethical dilemma for pharmaceutical companies. While companies may want to undertake and report these trials in an ethical manner, negative results can significantly affect product sales. There is accumulating evidence that company-financed trials are biased in favor of the product that the company makes. Ethical conduct in this article is defined as whether the trials are conducted in the best interests of the participants and/or reported in the best interests of patients. Nine examples of how clinical trials are violating multiple articles in the Declaration of Helsinki are discussed using concrete case reports from the literature. The recognition of ethical problems in company run trials is not something new, but to date no meaningful action has been taken to resolve this issue. What is necessary is to separate the financing of clinical trials from their conduct.

## Background

Clinical trials form the basis for medical practice. Regulators such as the Food and Drug Administration (FDA) use them to decide whether or not to approve a new drug. Doctors may not read the original research but that research is incorporated into continuing medical education talks, clinical guidelines and review articles. Pharmaceutical companies fund the vast majority of clinical trials [1] but these trials also present an economic and ethical dilemma to companies. Trials using rigorous methodology, the appropriate population groups and that are properly analyzed and published in a manner that accurately and completely presents their conclusions are the ideal. At the same time, companies need trials with positive results in order to get their drugs on the market and drive sales. Negative trials can have significant adverse effects on sales. Within one year of the publication of the Women’s Health Initiative trial that found that the estrogen/progestin combination caused an increased risk of cardiovascular disease and breast cancer in postmenopausal women, prescriptions for Prempro, the most widely sold estrogen/progestin combination, had declined by 66% in the United States (US) [2].

Therefore, there is a strong temptation to deviate from the ideal for economic reasons. The result is trials that are unethical in some manner. Here the term ethical is defined as whether the trials are conducted in the best interests of the participants and/or reported in the best interests of patients. The contention is not that every industry-funded trial is unethical, but the accumulating evidence provides strong grounds to believe that unethical behavior is not uncommon. A recent Cochrane review examined the association between the source of funding for trials and their results and conclusions. The authors found that the odds ratio for positive outcomes for drug studies sponsored by the manufacturing company was 2.15 (95% CI: 1.70 to 2.72) for results and 2.67 (95% CI 2.02 to 3.53) for conclusions [3]. Two separate groups have looked at clinical trials submitted to the FDA and then whether they were subsequently published and how closely the results in the FDA submissions matched those in the published versions. In both cases, positive trials were much more likely to be published and trials that had negative results when they were reviewed by the FDA had positive results in journal publications [4,5].

The accumulating evidence points to unethical behavior at all stages of clinical trials from their design, to whether or not ethical approval is obtained, the way that the trials are conducted, the use of ghost writers to write up the results, the withholding of data and finally the use of Phase IV trials as marketing tools. Even more importantly, is the violation of multiple articles about the ethical conduct of clinical trials that are enunciated in the Declaration of Helsinki [6].

### Main text

Declaration of Helsinki – articles 17, 18 and 24: “Medical research involving a disadvantaged or vulnerable population or community is only justified if the research is responsive to the health needs and priorities of this population or community…”; “Every medical research study involving human subjects must be preceded by careful assessment of predictable risks and burdens to the individuals and communities involved in the research …”; “…After ensuring that the potential subject has understood the information, the physician or another appropriately qualified individual must then seek the potential subject’s freely-given informed consent, preferably in writing. If the consent cannot be expressed in writing, the non-written consent must be formally documented and witnessed”.

Perhaps the most widely documented case of unethical behavior is the trial that Pfizer conducted with its antibiotic trovafloxacin (Trovan) on children with meningitis in Nigeria in 1996. As Stephens [7] points out, whereas planning for the trial would have taken about a year or longer in the United States, the trial in Nigeria was organized in just 6 weeks. Trovafloxacin not only had never been used before in the treatment of meningitis but it was being administered orally, a highly unusual way of treating meningitis. Furthermore, there was already a proven effective treatment for meningitis in this type of resource poor environment, intramuscular chloramphenicol that was, in fact, being used by Médecins sans Frontières at a nearby clinic to also treat children ill from the same meningitis outbreak. It later also turned out that the letter granting ethics approval was backdated and the local ethics committee had been set up a year after the trial [7,8]. A second, more recent example of the violation of article 17 comes from trials of olmesarten (Olmetec) between 2003–2006. Olmesarten is a medication for the treatment of hypertension, but these trials included children between the ages of 1–16 years of age. While there might have been a justification for treating children this young for high blood pressure none was provided by the sponsor (Daiichi Sankyo) [9].

Declaration of Helsinki – article 32: “The benefits, risks, burdens and effectiveness of a new intervention must be tested against those of the best current proven intervention,”

Both aripiprazole (Abilify) and quetiapine (Seroquel) were tested against placebo in patients with schizophrenia despite the risks of a relapse while on placebo. What is especially troubling about the quetiapine trials was that they were only being conducted to test different formulations of the same drug. While article 32 does contain exceptions for the use of placebos these do not apply in these cases [9].

Declaration of Helsinki – article 33: “At the conclusion of the study, patients entered into the study are entitled to be informed about the outcome of the study…”

Psaty and Rennie have documented a number of instances where clinical trials have been stopped for commercial, as opposed to medical, reasons. In discussing the early termination of a trial comparing verapamil with either a beta-blocker or a diuretic in the treatment of hypertension, they note “the responsible conduct of medical research involves a social duty and a moral responsibility that transcends quarterly business plans” [10]. Clearly, if trials are stopped before completion, then there will not be any outcomes related to safety or efficacy to convey to patients.

Declaration of Helsinki – article 30: “Authors, editors and publishers all have ethical obligations with regard to the publication of the results of research. Authors have a duty to make publicly available the results of their research on human subjects and are accountable for the completeness and accuracy of their reports. They should adhere to accepted guidelines for ethical reporting…”

Article 30 does not explicitly mention the obligation of companies to ensure that trials are fully and accurately reported but since it is the companies that control the data in the trials that they have funded, on face value article 30 should equally apply to them. There are a number of instances where companies have been guilty of withholding data from investigators and from those conducting systematic reviews. Full results of a trial looking at whether adding HIV-1 Immunogen to the treatment of AIDS patients would increase the efficacy of antiretrovirals could not be published because the sponsor, Remune, would not make the complete data set available to the investigators [11]. Whether or not oseltamivir (Tamiflu) lowers serious complications of influenza, such as pneumonia, is still unknown more than 3 years after Cochrane reviewers started trying to collect data for a systematic review to answer the question. Despite initial promises by Roche to provide full access to the data from all of the clinical trials, the company ultimately refused to do so citing a variety of reasons including confidentiality and the fact that there was another ongoing systematic review [12].

Besides the withholding of data, the practice of ghost writing also violates article 30. Ghost writing is the practice whereby companies hire professional medical writers to draft a manuscript that is ultimately signed by an academic and subsequently appears without an acknowledgement of the role of ghost writer. The obvious concern is that the article is drafted by someone who did not participate in the gathering or interpretation of the data and the message in the article is one that has been developed by the company to further its commercial interests. Surveys have estimated the prevalence of ghost writing is anywhere between 12% [13] and 75% in industry-initiated trials [14]. One example of how ghost writing distorts the medical literature is provided by Amsterdam and McHenry in their account of the paroxetine 352 study for the treatment for patients with bipolar type I major depressive disorder [15]. “The paroxetine 352 study manuscript was ghostwritten by Scientific Therapeutic Information, Inc. (STI) with funds provided by GSK; however, neither STI, the ghostwriter, nor GSK’s role in the production of the manuscript was acknowledged in the published article.” Although the primary outcome measure, whether paroxetine or imipramine was superior to placebo, was negative, the published article concluded that both drugs were efficacious in a *post hoc* subgroup.

Declaration of Helsinki – article 21: “Medical research involving human subjects may only be conducted if the importance of the objective outweighs the inherent risks and burdens to the research subjects.”

Phase IV trials are those undertaken once the drug is on the market. These trials are often undertaken not for scientific reasons but in order to enhance sales, so-called “seeding trials”. One anonymous former industry employee, writing in the BMJ, talked about how some of the studies he oversaw were designed to support a marketing message, e.g., to show a questionable advantage over a competitor or to increase the awareness of the condition within the medical community, not to evaluate the overall benefit:harm ratio of the drug [16]. The latest example of this practice is the multiple studies undertaken, mostly in developing countries, of the new insulin analogues. These trials had limited scientific value but enormous commercial value in promoting the wider use of more expensive insulin products [17].

## Discussion and conclusion

Documentation of these and other abuses is not something new. Bero and Rennie back in 1996 showed that industry funding negatively affected the content and quality of drug studies [18]. The persistence of unethical practices for at least a decade after the Bero and Rennie article appeared is one piece of evidence that in the time period between the mid 1990s to the mid 2000s industry practices did not change. A second, even more compelling example that at least one company continued to behave unethically, despite promising to reform, is Pfizer’s actions in the mid 2000s. In January 2004 when Pfizer pleaded guilty to marketing gabapentin (Neurontin) for unapproved uses its lawyers assured the United States Attorney’s Office that this practice would stop. However, in 2009, Pfizer once again pleaded guilty to the same practice, this time regarding the marketing of valdecoxib (Bextra) [19]. Whether industry has reformed now will probably not become apparent for at least 5 years as evidence of unethical practice is typically slow to manifest and often relies on documents revealed through court cases.

The existence of unethical practices is a serious problem not only because of the way that patients enrolled in clinical trials are treated but because of their effects on the quality of care that patients ultimately receive. The solution is simple in concept but politically very unappealing to those with the power to implement it – a separation between the funding of clinical trials and their conduct, analysis and write up. The Drug Utilization Acts of 1975 and 1977 and the Drug Regulation Reforms Acts of 1978 and 1979 proposed that the responsibility for clinical testing be transferred to the federal government [20]. More recently, critics of the current system have advocated that companies wishing to conduct clinical trials continue to fund them but the money would go to an independent institution such as the National Institutes of Health that would organize and manage clinical trials and the data that comes out of them [21,22]. For the sake of patients the time has come to act on these recommendations.
